# In Vitro Hydrodynamic Evaluation of Pulmonary Expanded Polytetrafluoroethylene Valved Conduits

**DOI:** 10.1093/icvts/ivag072

**Published:** 2026-03-02

**Authors:** Shunsuke Matsushima, Mutsuki Noda, Sara Kubo, Hironaga Shiraki, Hironori Matsuhisa, Kenji Okada, Osamu Kawanami

**Affiliations:** Department of Cardiovascular Surgery, Kobe Children’s Hospital, Kobe 650-0047, Japan; Division of Cardiovascular Surgery, Department of Surgery, Kobe University Graduate School of Medicine, Kobe 650-0017, Japan; Department of Mechanical Engineering, University of Hyogo, Himeji 671-2280, Japan; Department of Cardiovascular Surgery, Kobe Children’s Hospital, Kobe 650-0047, Japan; Division of Cardiovascular Surgery, Department of Surgery, Kobe University Graduate School of Medicine, Kobe 650-0017, Japan; Division of Cardiovascular Surgery, Department of Surgery, Kobe University Graduate School of Medicine, Kobe 650-0017, Japan; Department of Cardiovascular Surgery, Kobe Children’s Hospital, Kobe 650-0047, Japan; Division of Cardiovascular Surgery, Department of Surgery, Kobe University Graduate School of Medicine, Kobe 650-0017, Japan; Department of Mechanical Engineering, University of Hyogo, Himeji 671-2280, Japan; Advanced Medical Engineering Research Institute, University of Hyogo, Himeji 670-0836 Japan

**Keywords:** right ventricular outflow tract reconstruction, pulmonary valve replacement, expanded polytetrafluoroethylene conduit, handmade valve, in vitro experiment, flow visualization, particle image velocimetry

## Abstract

**Objectives:**

Various designs are proposed for pulmonary expanded polytetrafluoroethylene (ePTFE) conduits and have been applied in clinical practice. However, experimental data to support them are limited. We conducted an in vitro experiment using a circulatory simulator to evaluate their haemodynamic performance and hydrodynamic characteristics.

**Methods:**

Three root models with a 24-mm basal ring (A, straight; B, with small sinuses; C, with large sinuses) were 3D-printed. Cusps were uniformly cut out from a 0.1-mm-thick ePTFE membrane and sewn to the inter wall of the models. Model A had a single suture on the free margins near the commissures. Each model was tested with a pump size of 70 mL, 70 beats/min, and arterial pressure of 30/10 mmHg. The valve behaviour was recorded by a high-speed camera, and particle image velocimetry (PIV) was performed in the region behind the model housing section.

**Results:**

Peak transvalvular pressure gradients were 4.0, 4.8, and 4.3 mmHg (*P* = .95), and geometric orifice areas were 2.34, 2.38, and 2.46 cm^2^ (*P* = .96) in models A, B, and C, respectively. Particle image velocimetry revealed peak instantaneous velocity was 1.69, 1.69, and 1.65 m/s (*P* = .74) and peak Reynolds shear stress in the midsystolic phase was 56.8, 49.5, and 25.5 Pa (*P* = .05) in models A, B, and C, respectively. Model C tended to have a lower distribution of turbulent flow than the other models.

**Conclusions:**

All models exhibited sufficient opening and acceptable Reynolds shear stress values. The sinus contributed to the suppression of turbulent flow, which may lead to an improvement of conduit durability, but its effect was dependent on the sinus size.

## Graphical Abstract

**Figure:**
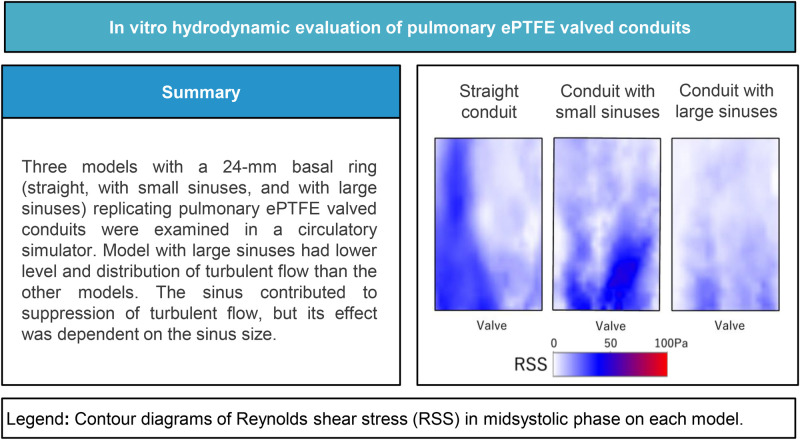

## INTRODUCTION

Pulmonary valve replacement or right ventricle-pulmonary artery conduit implantation are essential for repairing various congenital heart defects. While homografts and bioprostheses, including bovine jugular vein conduits and aortic valve prostheses are often used,^1^^,^^2^ expanded polytetrafluoroethylene (ePTFE) has emerged as the material of choice for pulmonary valved conduits.^3–9^ Handmade or commercialized ePTFE conduits have already been applied in clinical practice and provided favourable durability.^4–9^ Their clinical advantages and the easy availability of ePTFE facilitate the spread of handmade ePTFE valved conduits to different institutes.

Various designs are proposed for handmade ePTFE valved conduits. Most reported literature, including ours involves the use of a straight tube and a 0.1-mm-thick membrane for their root and cusp, respectively.^5–7^ A piece of connected 3 cusps is cut out from the ePTFE membrane and sewn to the inner wall of the inverted ePTFE tube. This valved conduit does not need any additional product processing and can be hand-crafted in the same operation room. Meanwhile, handmade ePTFE conduits with bulging sinuses and a fan-shaped valve have gained popularity, especially in Japan.^3^^,^^4^ They require special manufacturing, such as thermal processing, leading to their limited global use, but their structures, like a normal semilunar valve are supposed to contribute to better pulmonary valve function.

However, these handmade ePTFE valved conduits have been introduced to real patients by necessity, mainly in countries where homograft availability was limited. Therefore, experimental data supporting them are quite limited, and the difference of their designs in haemodynamic performance has been inadequately tested.^9–12^ Scientific evidence justifying the new valve substitutes should be accumulated for ethical means, and the comparative study of each design is hoped to invent better alternatives. We have developed an in vitro experiment using a pulmonary circulatory simulator for assessing our handmade ePTFE valved conduit.^9^ In addition to conventional haemodynamic assessment, hydrodynamic characterization by flow visualization and particle image velocimetry (PIV) is included to obtain more accurate information on valve function.^9^^,^^12–16^ We believe that this experimental method could explain the functional implication of each conduit design in detail.

This study described the experimental characteristics of conduits having a hand-sewn tricuspid valve, with or without bulging sinuses, and explored the role of the sinuses of Valsalva on the opening of pulmonary valved conduits.

## METHODS

### Model construction

**Figure 1.**
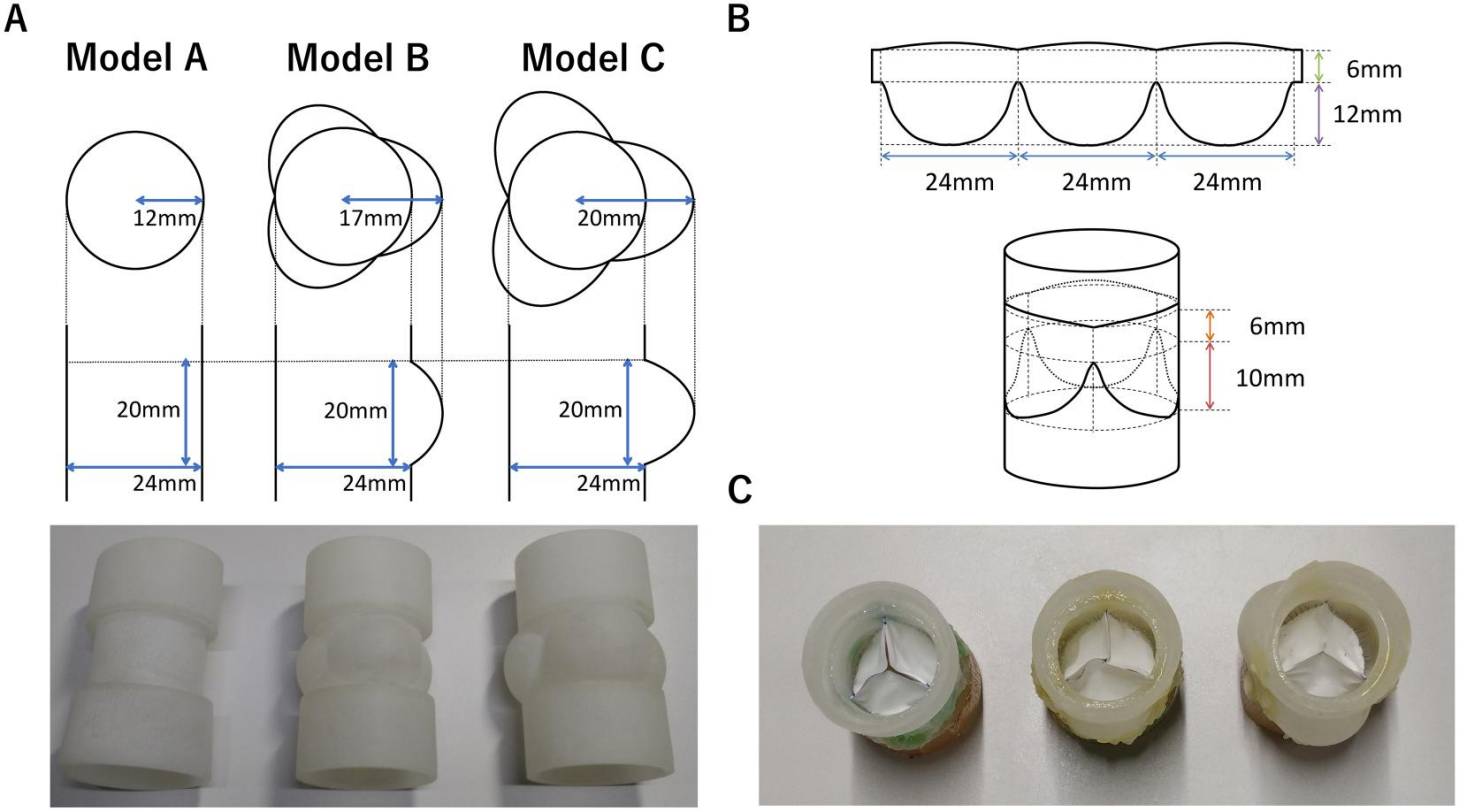
Model Design. (A) Root Design. (B) Cusp Design. (C) Internal Views of the Closed Position.

No ethical approval was required for this study, as it did not involve human or animal subjects. Three root designs composed of straight (model A), with small bulging sinuses (model B), and with large bulging sinuses (model C) were prepared. Their basal rings were all 24 mm, and the details of their designs are presented in Figure 1A. Large bulging sinuses of model C were sized according to the normal sinus of Valsalva of the aortic valve,^17^^,^^18^ as the geometry of the normal pulmonary valve has not been well studied and changes significantly during the cardiac cycle.^19^ The small bulging sinuses of model B were set to the mid-range value between model A and C. Root models were 3D-printed with soft silicon rubber (AR-G1L; KEYENCE Corp.) by a high-resolution inkjet 3D printer (AGILISTA-3200; KEYENCE Corp.). Cusps were cut out uniformly from a 0.1-mm thick ePTFE membrane (GoreTex; W.L. Gore & Associates) and sewn to the inner wall of root models along the cusp design of our 24-mm conduits (Figure 1B and C).^6^ In model A replicating our 24-mm conduits, a single suture was placed on the free margins near the commissures to prevent the cusp from sticking, as previously published.^5–7^ In order to show the reproducibility of these handmade valved conduits, 3 specimens for each model were prepared. A total of 9 specimens were crafted by a single surgeon (S.M.) and examined in a pulmonary circulatory simulator.

### Pulmonary circulatory simulator

**Figure 2.**
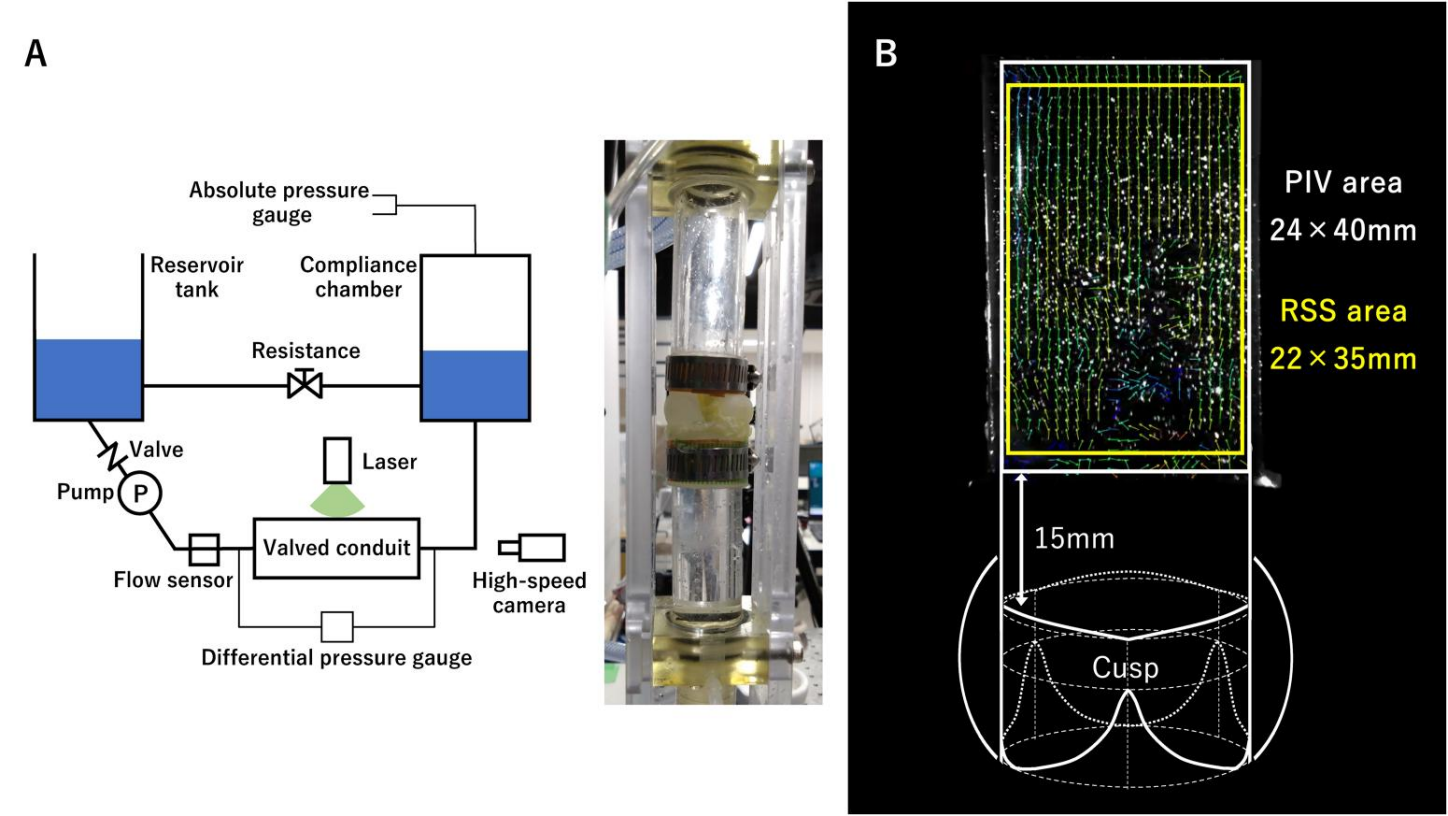
Schema and Picture of the in Vitro Experiment. (A) Schema of the Simulator and Picture of the Model Housing Section. (B) Area Analysed Using Particle Image Velocimetry and Area Used for Reynolds Shear Stress (RSS) Calculation.

Our pulmonary circulatory simulator was composed of a bladder pump with an inlet valve (VCT-50χ; NIPRO Corp.), a model housing section, a compliance chamber, a vascular resistance unit, and a venous reservoir tank (Figure 2A).^9^ An ultrasound flow sensor (ME16PXL; Transonic Systems Inc.) was set upstream of the model housing section. The absolute pressure in the compliance chamber and the differential pressure from the inlet to the outlet of the model housing section were measured by pressure transducers. The working fluid was 36% glycerine by volume in water to simulate the kinematic viscosity of blood.^9^^,^^15^^,^^16^ Arterial pressure was adjusted to 30/10 mmHg with a pump size of 70 mL and 70 beats/min. The ratio of systolic to diastolic duration was set to two-thirds. Each specimen was examined during 1 stabilized run under highly controlled and fixed geometric, fluid, and haemodynamic conditions, ie, each model was assessed with 3 runs of the experiments.

### Haemodynamic assessment

To observe the cusp motions during the entire cycle, the valve behaviour was recorded by an en face high-speed camera (High-Speed Camera K9-USB; KATO Koken Co., Ltd.) at 1000 fps, and the geometric orifice area (GOA) was analysed by thresholding in ImageJ/Fiji (https://imagej.net/software/fiji/). Stroke volume, closing volume, regurgitant fraction (integrating closing volume and transvalvular leakage), and peak transvalvular pressure gradient were calculated using an ultrasound flow sensor and pressure transducers as haemodynamic parameters.^9^^,^^15^^,^^16^ These data were represented as median values from 5 consecutive cycles for each root design.

### Hydrodynamic characterization

PIV was performed using a continuous-wave YVO4 laser (CW532-1WM; KANOMAX JAPAN Inc.) and 50-μm polyamide particles (ORGASOL; KANOMAX JAPAN Inc.) as the light source and visualization particles, respectively.^9^ In an acrylic pipe (ID, 24 mm) immediately behind the model housing section, the particles were filmed from the lateral view by the high-speed camera at 1500 fps, and a 24 × 40 mm rectangular area 15 mm above the cusp commissures was analysed with PIV software (FlowExpert2D3C-L; KATO Koken Co., Ltd.) (Figure 2B). In addition to the flow visualization, the Reynolds shear stress (RSS, Pa) in the midsystolic phase was derived from the images between the first and third quarters of the systolic phase according to the following equation:


$$\mathrm{RSS}=\rho \sqrt{{\left(\frac{\bar{u^{\prime }u^{\prime }}-\bar{v^{\prime }v^{\prime }}}{2}\right)}^{2}+{\left(\bar{u^{\prime }v^{\prime }}\right)}^{2}}$$

where ρ is the density (kg/m^3^) of the working fluid and $u^{\prime }$ and $v^{\prime }$ are the x and y fluctuation components of the velocity vector (m/s), respectively.^9^^,^^14^^,^^15^ Peak RSS values were obtained from a 22 × 35 mm rectangular area of each contour diagram and represented with median values of 3 models for each root design (Figure 2B).

### Statistical analysis

All statistical calculations were performed with the R environment (version 4.0.3; R Foundation). Continuous variables were expressed as medians, and comparisons between models were done using the Kruskal–Wallis test. Contour diagrams of RSS were described by the heatmaps function of the Plotly’s R graphing library (https://plotly.com/r/). The curves of GOAs were drawn with Microsoft Office Excel (version 2021; Microsoft Corp.).

## RESULTS

### Haemodynamic performance

**Figure 3.**
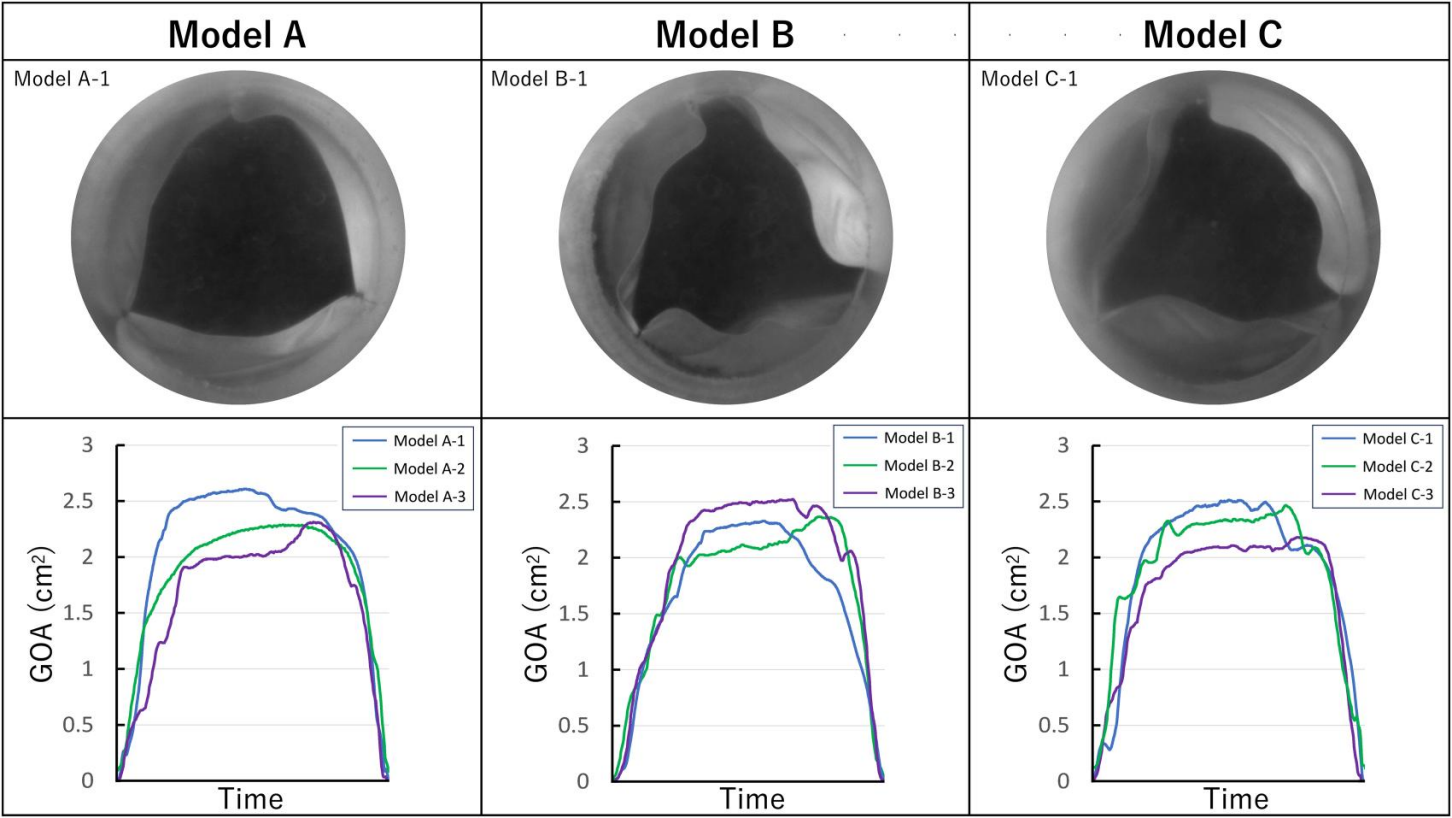
En Face Imaging of the Valve Behaviour. GOA, Geometric Orifice Area.

The haemodynamic parameters obtained from the analyses are listed in Table 1. Closing volume was a little lower in model C, and regurgitation fraction was a little higher in model A than in other models, but all of them did not reach statistical significance. High-speed imaging of the valve behaviour is presented in Video 1 and summarized in Figure 3. The maximum geometric orifice areas of models A, B, and C were 2.34, 2.38, and 2.46 cm^2^, respectively (*P* = .96). While these values showed no significant difference, cusp-free margins in midsystole appeared to be tightened in model A and wrinkled in models B and C.

**Table 1.** Haemodynamic Parameters

Model	Stoke volume (mL)	Closing volume (mL)	Regurgitant fraction (%)	Peak pressure gradient (mmHg)
A (*n* = 3)	76.3	7.6	14.0	4.0
B (*n* = 3)	76.0	6.1	11.6	4.8
C (*n* = 3)	76.4	5.2	12.1	4.3
*P*	.73	.30	.06	.95

Values are presented as median.

### Hydrodynamic characteristics

**Figure 4.**
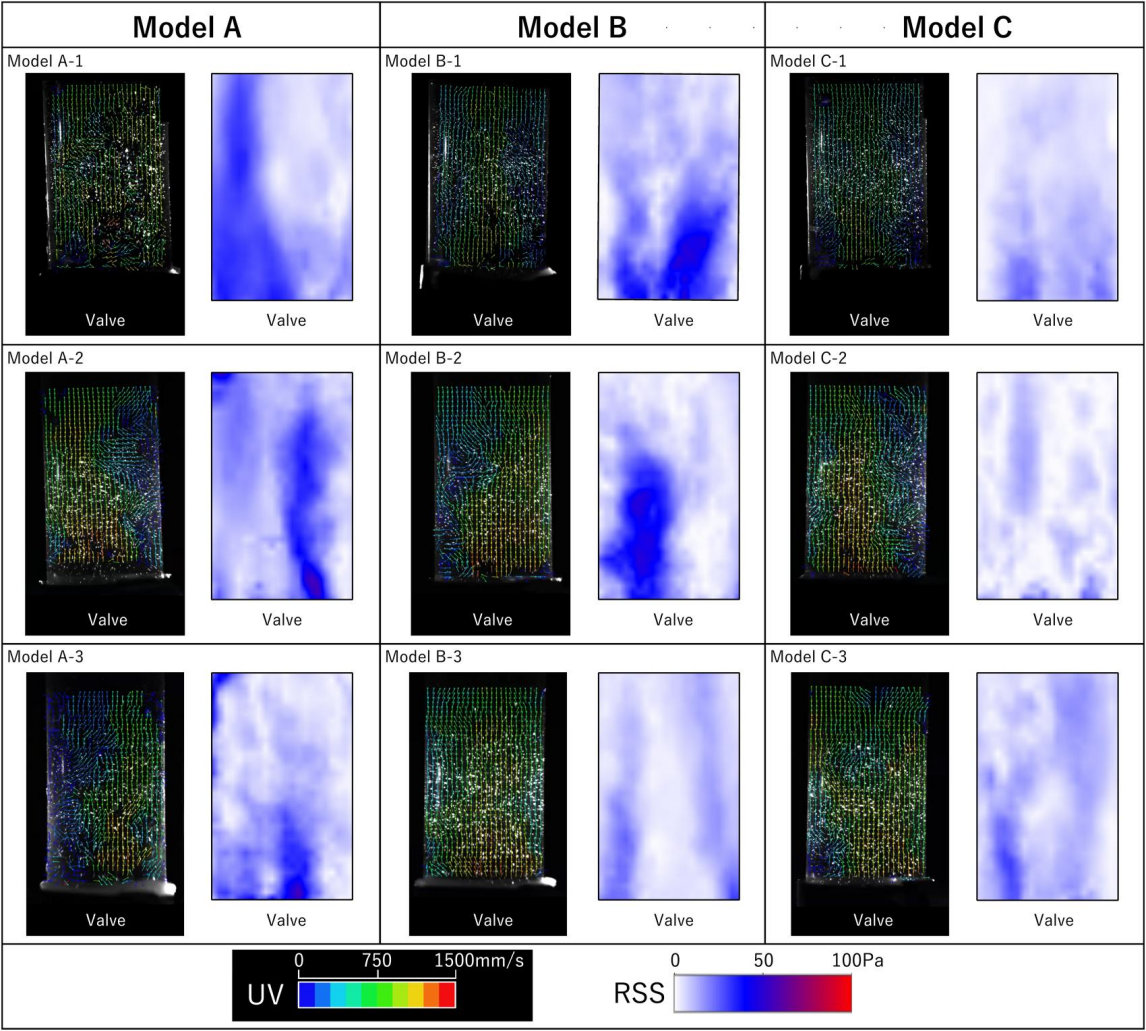
Velocity Maps and Contour Diagrams of Reynolds Shear Stress (RSS) in Midsystolic Phase on Each Model.

Flow visualization using PIV and contour diagrams of the RSS in the midsystolic phase are shown in Video 1 and Figure 4, respectively. The peak instantaneous velocity was 1.69, 1.69, and 1.65 m/s in models A, B, and C, respectively (*P* = .74). The peak instantaneous velocity in the blood flow direction was 1.23, 1.24, and 1.23 m/s in models A, B, and C, respectively (*P* = .57). The peak instantaneous velocity in the orthogonal direction was 1.23 m/s in each model. The peak RSS in the midsystolic phase was 56.8, 49.5, and 25.5 Pa in models A, B, and C, respectively (*P* = .05), and model C tended to have a lower distribution of turbulent flow than the other models.

## DISCUSSION

The presence of bulging sinuses is an obvious structural difference between handmade ePTFE valved conduits implanted in actual patients. In the aortic position, the role of the sinus of Valsalva has been explored extensively using various experimental modalities; the sinus prevents occlusion of coronary arteries and creates efficient cusp opening and smooth cusp closing.^16^^,^^20–23^ Its positive effect, mainly on the opening process of the valve has been confirmed clinically.^24^^,^^25^ It is reasonable that the right ventricular outflow tract should also be reconstructed in a fashion similar to that of the normal pulmonary valve, and the clinical outcomes of handmade ePTFE valved conduits with bulging sinuses are favourable.^3^^,^^4^ Nonetheless, straight ePTFE valved conduits have shown comparable durability, which results in an increasing number of straight conduit implantation.^5–7^ This is because a variety of operative factors, such as patients’ size, conduit size selection, irregular anastomotic sites, and orthotopic/heterotopic implantation, obscure the clinical benefit of bulging sinuses. To clarify the function of the sinus in the pulmonary position, this study compared models replicating each conduit design under the same condition in the pulmonary circulatory simulator. Our in vitro experiment, including high-speed imaging and flow visualization can provide more detailed and realistic evidence of semilunar valve function than in vivo examination and computer simulation and complement them in configuring conditions and interpreting the results.

All examined models had satisfactory opening performance with sufficient GOA and low transvalvular pressure gradient. Clinical routine examinations such as echocardiography and cardiac catheterization must report the same results between their designs, which can support the continued use of straight-valved conduits. However, the present hydrodynamic evaluation revealed detailed velocity vectors of blood flow generated by each root design and their different turbulence. To assess its influence objectively, we calculated RSS values that are known to correlate with platelet activation and haemolysis.^13^ Its thresholds for platelet activation and haemolysis vary depending on reports but are roughly considered to be 100 and 3000 Pa, respectively.^12^^,^^14^^,^^15^^,^^26^ Although the available literature about its normal range in the pulmonary site is quite limited,^13^ the peak RSS magnitudes of adult-sized pulmonary ePTFE conduits and aortic valve prostheses have been regarded as 50 and 100-350 Pa, respectively.^12^^,^^15^ While RSS values were acceptable as a pulmonary valve substitute in all models, model C with large bulging sinuses had a lower level and distribution of turbulent flow in the downstream position than the other models. The detailed mechanisms by which bulging sinuses contribute to valve opening are lesser known in comparison with valve closing.^21^^,^^22^ Probably, vortex rings formed at the sinuses would help cusps open sufficiently and move smoothly along pulsatile blood flow from the pump and keep its flow laminarity.^10^^,^^16^^,^^20^^,^^22–25^ This suppression of turbulent flow may lead to improvement of conduit durability by reducing platelet activation and inflammatory reaction.^13^^,^^15^ Additional stitches to the cusps near the commissures in model A is for simulating clinically common straight ePTFE valved conduits and can cause the stretch of its RSS value by restricting the flow pathway, but model B without these stitches also triggered similar turbulence despite the presence of bulging sinuses. Specifically, the benefit of the bulging sinus for valve opening was dependent on its size, which appears to require a radius about 1.6 times larger in analogy to the normal aortic valve.^17^^,^^18^ Opposite flow generation following the ejected flow, leading to vortex flow, may need adequate entry and space between the cusps and conduit wall.

In terms of closing characteristics, this study did not indicate an apparent distinction between models, as previous reports have suggested.^10^^,^^20–22^^,^^24^ In a computer simulation presenting aortic valve cusp motion with or without the sinus of Valsalva clearly, the sinus facilitated its smooth closure and reduced abnormal stress on the cusp.^21^ It is assumed that vortex formation in the sinus should create this effect by relieving cusp deformation and extending slow closing distance.^20^^,^^21^^,^^24^ These mechanisms may explain the reduction of the closing volume with bulging sinuses, but there was no significant difference in both the present experiment and other reports.^22^ We hope that our future in vitro experiments will settle this argument by repeating examinations of various root models and analysing PIV data during the late phase of ejection and the early phase of closure.

Following these results, we still use our straight ePTFE valved conduits in paediatric patients because we value the actual size of the blood flow pathway and have implanted larger valved conduits than other institutions.^4^^,^^6^^,^^7^ For example, the 24 mm straight conduit can be implanted in patients weighing ≥ 25 kg and is supported to function with a peak velocity < 3.0 m/s in patients with a body weight of up to 75 kg and a body surface area of up to 2.0 m^2^ for more than 12 years postoperatively.^6^ If a 24 mm conduit would benefit from bulging sinuses, the conduit width at the sinus level should reach approximately 32 mm,^17^^,^^18^ which is too large to implant in patients weighing 25 kg. Therefore, 24 mm conduits with bulging sinuses will be the best option in adult-sized patients weighing ≥ 50 kg as the group using them reported.^4^ If the positive effect on the closing process would be demonstrated even in conduits with small bulging sinuses, this context and our policy should be revised.

### Limitations

This study had several limitations. Soft silicone rubber was used for root models to reproduce bulging sinuses precisely with the 3D printer. Its thickness and elasticity are different from the ePTFE standard wall and may have contributed to more regurgitant fraction than expected from our clinical experience.^6^ The area analysed by the PIV was 15 mm apart from above the valve due to the opacity of root models and metal hose clamps, and the curved right ventricular outflow tract and branch pulmonary arteries were not installed in the simulator, both of which can blur turbulent flow generated by the valve.^13^ In addition, the PIV was executed in a single plane and required multidirectional projection for a more detailed analysis. Finally, only a small number of models with the same size under the same cardiac output were examined, and 1 stabilized experimental run per specimen was evaluated.

## CONCLUSION

Our in vitro experiment using a circulatory simulator can evaluate semilunar valve function in detail by visualizing cusp movement and each velocity of generated blood flow. Regardless of whether bulging sinuses exist, all examined models had satisfactory haemodynamic performance and hydrodynamic characteristics with sufficient opening and acceptable RSS values as a pulmonary valve substitute. The presence of bulging sinuses contributed to suppression of turbulent flow, which may lead to improvement of conduit durability, but its effect was dependent on the sinus size.

## Data Availability

The data underlying this article will be shared on reasonable request to the corresponding author.

## References

[1] Willetts RG, Stickley J, Drury NE, et al Four right ventricle to pulmonary artery conduit types. J Thorac Cardiovasc Surg. 2021;162:1324-1333.e3. 33640135 10.1016/j.jtcvs.2020.12.144

[2] Boethig D, Avsar M, Bauer UMM, et al; National Register For Congenital Heart Defects Investigators. Pulmonary valve prostheses: patient’s lifetime procedure load and durability. Evaluation of the German national register for congenital heart defects. Interact CardioVasc Thorac Surg. 2022;34:297-306. 34436589 10.1093/icvts/ivab233 PMC8929479

[3] Yamagishi M. Right ventricular outflow reconstruction using a polytetrafluoroethylene conduit with bulging sinuses and tricuspid fan-shaped polytetrafluoroethylene valve. Oper Tech Thorac Cardiovasc Surg. 2016;21:211-229.

[4] Hongu H, Yamagishi M, Maeda Y, et al Expanded polytetrafluoroethylene conduits with bulging sinuses and a fan-shaped valve in right ventricular outflow tract reconstruction. Semin Thorac Cardiovasc Surg. 2022;34:972-980. 33691193 10.1053/j.semtcvs.2021.02.026

[5] Ootaki Y, Muralidaran A, Mehta I, Walsh MJ, Ungerleider RM. Long-term outcomes with expanded polytetrafluoroethylene valved conduits in pediatric patients. Ann Thorac Surg Short Rep. 2024;2:810-814. 39790602 10.1016/j.atssr.2024.04.021 PMC11708714

[6] Matsushima S, Takahashi R, Kubo S, Higashida A, Oshima Y, Matsuhisa H. Pulmonary expanded polytetrafluoroethylene conduits with a hand-sewn tricuspid valve. Interdiscip Cardiovasc Thorac Surg. 2025;40:ivaf020. 10.1093/icvts/ivaf020 PMC11997764 39909862

[7] Shi Q, Shan Y, Chen G, et al Midterm outcomes for polytetrafluoroethylene valved conduits. Ann Thorac Surg. 2022;114:1778-1785. 34717907 10.1016/j.athoracsur.2021.09.051

[8] Baird CW, Chávez M, Backer CL, Galantowicz ME, Del Nido PJ. Preliminary results with a novel expanded polytetrafluoroethylene-based pulmonary valved conduit. Ann Thorac Surg. 2022;114:2314-2321. 34838744 10.1016/j.athoracsur.2021.10.033

[9] Matsushima S, Matsuhisa H, Wakita K, et al Expanded polytetrafluoroethylene conduits with curved and handsewn bileaflet designs for right ventricular outflow tract reconstruction. J Thorac Cardiovasc Surg. 2024;167:439-449.e6. 37356475 10.1016/j.jtcvs.2023.05.043

[10] Suzuki I, Shiraishi Y, Yabe S, et al Engineering analysis of the effects of bulging sinuses in a newly designed pediatric pulmonary heart valve on hemodynamic function. J Artif Organs. 2012;15:49-56. 21956206 10.1007/s10047-011-0609-1

[11] Chang TI, Hsu KH, Luo CW, Yen JH, Lu PC, Chang CI. In vitro study of trileaflet polytetrafluoroethylene conduit and its valve-in-valve transformation. Interact CardioVasc Thorac Surg. 2020;30:408-416. 31899505 10.1093/icvts/ivz274

[12] Zhu G, Wei Y, Yuan Q, Cai L, Nakao M, Yeo JH. In vitro assessment of the impacts of leaflet design on the hemodynamic characteristics of ePTFE pulmonary prosthetic valves. Front Bioeng Biotechnol. 2019;7:477. 32076599 10.3389/fbioe.2019.00477 PMC7006451

[13] Stein PD, Walburn FJ, Sabbah HN. Turbulent stresses in the region of aortic and pulmonary valves. J Biomech Eng. 1982;104:238-244. 7120950 10.1115/1.3138355

[14] Raghav V, Sastry S, Saikrishnan N. Experimental assessment of flow fields associated with heart valve prostheses using particle image velocimetry (PIV): recommendations for best practices. Cardiovasc Eng Technol. 2018;9:273-287. 29532332 10.1007/s13239-018-0348-z

[15] Hatoum H, Yousefi A, Lilly S, Maureira P, Crestanello J, Dasi LP. An in vitro evaluation of turbulence after transcatheter aortic valve implantation. J Thorac Cardiovasc Surg. 2018;156:1837-1848. 29961588 10.1016/j.jtcvs.2018.05.042 PMC6196367

[16] Sadri V, Madukauwa-David ID, Yoganathan AP. In vitro evaluation of a new aortic valved conduit. J Thorac Cardiovasc Surg. 2021;161:581-590.e6. 31879167 10.1016/j.jtcvs.2019.09.181

[17] Izawa Y, Mori S, Tretter JT, et al Normative aortic valvar measurements in adults using cardiac computed tomography - a potential guide to further sophisticate aortic valve-sparing surgery. Circ J. 2021;85:1059-1067. 33408304 10.1253/circj.CJ-20-0938

[18] Jelenc M, Jelenc B, Poglajen G, Lakič N. Aortic valve leaflet and root dimensions in normal tricuspid aortic valves: a computed tomography study. J Card Surg. 2022;37:2350-2357. 35526127 10.1111/jocs.16587

[19] Jelenc M, Jelenc B, Habjan S, et al Comparison of pulmonary and aortic root and cusp dimensions in normal adults using computed tomography: potential implications for ross procedure planning. Interdiscip Cardiovasc Thorac Surg. 2024;39:ivae206. 10.1093/icvts/ivae206 PMC11665635 39657909

[20] Fries R, Graeter T, Aicher D, et al In vitro comparison of aortic valve movement after valve-preserving aortic replacement. J Thorac Cardiovasc Surg. 2006;132:32-37. 16798299 10.1016/j.jtcvs.2006.02.034

[21] Katayama S, Umetani N, Sugiura S, Hisada T. The sinus of Valsalva relieves abnormal stress on aortic valve leaflets by facilitating smooth closure. J Thorac Cardiovasc Surg. 2008;136:1528-1535, 1535.e1. 19114202 10.1016/j.jtcvs.2008.05.054

[22] Toninato R, Salmon J, Susin FM, Ducci A, Burriesci G. Physiological vortices in the sinuses of Valsalva: an in vitro approach for bio-prosthetic valves. J Biomech. 2016;49:2635-2643. 27282961 10.1016/j.jbiomech.2016.05.027 PMC5061069

[23] Salica A, Pisani G, Morbiducci U, et al The combined role of sinuses of Valsalva and flow pulsatility improves energy loss of the aortic valve. Eur J Cardiothorac Surg. 2016;49:1222-1227. 26362428 10.1093/ejcts/ezv311

[24] Leyh RG, Schmidtke C, Sievers HH, Yacoub MH. Opening and closing characteristics of the aortic valve after different types of valve-preserving surgery. Circulation. 1999;100:2153-2160. 10571974 10.1161/01.cir.100.21.2153

[25] Matsumori M, Tanaka H, Kawanishi Y, et al Comparison of distensibility of the aortic root and cusp motion after aortic root replacement with two reimplantation techniques: Valsalva graft versus tube graft. Interact CardioVasc Thorac Surg. 2007;6:177-181. 17669804 10.1510/icvts.2006.143289

[26] Jhun CS, Stauffer MA, Reibson JD, et al Determination of Reynolds shear stress level for hemolysis. Asaio J. 2018;64:63-69. 28661910 10.1097/MAT.0000000000000615 PMC5732101

